# Risk of malignancy in cytologically indeterminate thyroid nodules harboring thyroid stimulating hormone receptor mutations

**DOI:** 10.3389/fendo.2022.1073592

**Published:** 2022-12-22

**Authors:** Dorota Whitmer, John E. Phay, Shelby Holt, Benjamin O’Donnell, Jay Nguyen, Dennis Joseph, Anthony Chi, Shuyang Wu, Yangyang Hao, Jing Huang, Joshua P. Klopper, Richard T. Kloos, Giulia C. Kennedy, Joyce Shin

**Affiliations:** ^1^ Department of Endocrinology, Endocrinology and Metabolism Institute, Cleveland Clinic, Cleveland, OH, United States; ^2^ Department of Surgery, The James Comprehensive Cancer Center, The Ohio State University, Columbus, OH, United States; ^3^ Department of Surgery, UT Southwestern Medical Center, Dallas, TX, United States; ^4^ Wexner Medical Center, The Ohio State University, Columbus, OH, United States; ^5^ Lake Cumberland Regional Hospital, Somerset, KY, United States; ^6^ Endocrinology Center of Lake Cumberland, Somerset, KY, United States; ^7^ Department of Pathology, Mid-Atlantic Permanente Medical Group, Rockville, MD, United States; ^8^ 8Department of Research and Development, Veracyte, South San Francisco, CA, United States; ^9^ Department of Medical Affairs, Veracyte, South San Francisco, CA, United States; ^10^ Department of Clinical Affairs, Veracyte, South San Francisco, CA, United States; ^11^ Department of Endocrine Surgery, Endocrinology and Metabolism Institute, Cleveland Clinic, Cleveland, OH, United States

**Keywords:** thyroid nodule, molecular diagnosis, TSH receptor (TSHR), Afirma ^®^, thyroid cancer

## Abstract

**Objectives:**

To evaluate the frequency and risk of malignancy of TSHRpI568T mutations discovered in indeterminate thyroid nodules (ITN) within the Veracyte CLIA laboratory undergoing Afirma^®^ Genomic Sequencing Classifier (GSC) testing, and to evaluate a broader cohort of TSHR variants and their categorization as Afirma GSC benign (GSC-B) or suspicious (GSC-S). Finally, we seek to assess the risk of malignancy (ROM) of this group of TSHR mutated ITN in the GSC-S category.

**Methods:**

ITN submitted to Veracyte for Afirma GSC testing between October 2017 and February 2022 were analyzed for TSHR variants and rates of GSC-B and GSC-S were calculated based upon BIII or IV cytology, by TSHR variant codon amino acid (AA) substitution, age, and gender. For GSC-S samples, surgical pathology reports were requested, and the rate of malignancy was calculated.

**Results:**

Five percent of the ITN samples harbored an isolated TSHR variant and 5% of those were classified as GSC-S. Among TSHRpI568T samples, 96% were GSC-B and of the GSC-S samples, 21% were malignant. Among an unselected group of TSHR, absent TSHRpI568T mutations, 16.3% of GSC-S samples were malignant, all but one with codon mutations in the transmembrane subdomains of the TSHR. This prompted a dedicated evaluation of transmembrane codons which revealed a malignancy rate of 10.7% among GSC-S nodules. In total, 13/85 (15.3%) TSHR mutated ITN with Afirma GSC-S results were found to be malignant.

**Conclusions:**

TSHR variants are rare in ITN, and most are categorized as benign under Afirma GSC testing which carries a < 4% risk of malignancy. For GSC-S ITN with TSHR mutations, the risk of malignancy is ≥= 15%, which is clinically meaningful and may alter treatment or monitoring recommendations for patients.

## Introduction

The thyroid stimulating hormone receptor (TSHR) is a member of the large superfamily of G-protein-coupled receptors that is expressed primarily in thyroid follicular cells as well as non-thyroidal sites including fibroblasts, adipocytes, brain, and other sites (1–3). Structurally, the receptor has seven transmembrane domains with a long extracellular amino acid chain leading to the NH2-terminal peptide and a shorter intracellular chain leading to the carboxy terminus (4, 5). TSH binding of the receptor leads primarily to activation of adenylyl cyclase and a resultant cyclic AMP cascade that regulates growth, differentiation, and hormone secretion of thyroid follicular cells (3, 6).

Mutations of the TSHR have been associated most with thyroid autonomy and toxic adenomas (3, 7, 8). Though it is often taught that toxic thyroid nodules are benign, and hyperthyroidism is correlated with a low risk of thyroid cancer, several surgical series show a higher-than-expected incidence of thyroid cancer. Mohamed et al. reported a 21% malignancy rate in patients who underwent surgery with toxic multinodular goiter or toxic adenomas, though there was not specification of the presence of thyroid cancers within the autonomous thyroid tissue (9). Tam et al. found a similar 19% malignancy rate in toxic nodular goiter resections with specification that half (9.5%) of these malignancies arose from the autonomous nodule/s (10).

There have been at least two studies looking specifically at the clinicopathological correlation of TSHR variants in indeterminate thyroid nodules (ITN). Mon et al. evaluated 703 ITNs or nodules with benign cytology and suspicious clinical features with the Thyroseq^®^ v2 next generation sequencing (NGS) platform (11). TSHR variants were detected in 4.4% of thyroid nodules. Of 16 samples with known histopathology, 4 were malignant though one of those sample had a concurrent BRAFV600E mutation. Therefore, 3/15 (20%) ITN with an isolated TSHR variant were malignant. Interestingly, 2 of the 3 samples had initial benign cytology reads and three of those patients had a suppressed TSH. All were follicular thyroid carcinomas (FTC). Guan et al. reported on 388 ITN samples sent for Thyroseq v2 analysis and 4.7% of nodules were found to harbor TSHR variants (4). Of the 7 nodules that were resected, none were malignant.

The current study was influenced by an interesting case report of an aggressive thyroid malignancy with a TSHRpI568T mutation (12). Briefly, a 53-year-old female underwent a fine needle aspiration biopsy (FNAB) of a thyroid nodule with resultant Bethesda V cytology and Afirma^®^ Xpression Atlas (XA) testing that revealed a TSHRpI568T mutation (13). Based on ultrasound and clinical findings, a total thyroidectomy was performed and revealed a 2 cm papillary thyroid carcinoma with posterior invasion and with a need for a partial resection of 2 tracheal rings. As most literature describes TSHR mutated nodules as benign and when malignant, low risk, a query was made to Veracyte, Inc regarding the prevalence and risk of malignancy of TSHRpI568T mutated thyroid nodules (14).

The objectives of this study are to evaluate the frequency and risk of malignancy of TSHRpI568T mutations discovered in ITN within the Veracyte CLIA laboratory from FNAB samples undergoing Afirma Genomic Sequencing Classifier (GSC) testing, and to evaluate a broader cohort of TSHR variants and their categorization as Afirma GSC benign (GSC-B) or suspicious (GSC-S) (15). Finally, we seek to assess the risk of malignancy (ROM) of this group of TSHR mutated ITN in the GSC-S category.

## Materials and methods

Afirma testing analyzes aspirate material from ITN placed in an RNA protect reagent and processed as previously described (13, 15–17). Samples with isolated TSHR variants discovered by Afirma Xpression Atlas (XA) (which reports on 905 gene variants and 235 fusion pairs from RNA-sequencing of 593 genes) testing were evaluated in Afirma GSC-B and GSC-S samples across The Bethesda System for Reporting Thyroid Cytopathology categories III and IV (BIII and BIV) that were submitted to Veracyte as part of routine clinical care (13, 15, 18). The Afirma GSC utilizes an ensemble model of 1115 differentially expressed core genes, and over 10,000 genes in total, which manages the benign versus suspicious classifier categorization (15). XA identifies over 80 TSHR variants, including mutations at codons within the extracellular, transmembrane, and intracellular domains of the TSHR. The analyzed samples were collected between October 2017 and February 2022. As described in the Afirma XA analytical and clinical validation study, for research purposes only, molecular variants can be detected in the GSC-B category though these are not reported clinically (13).

The rates of GSC-B and GSC-S for TSHR variants were calculated based upon BIII or IV cytology, by TSHR variant codon amino acid (AA) substitution, age, and gender. For GSC-S samples, surgical pathology reports were requested under an IRB protocol (WCG IRB protocol # DHF 005-044) and the rate of malignancy was calculated. Non-invasive follicular neoplasm with papillary like nuclear features (NIFTP) was considered malignant as these lesions are not categorized histologically as benign and surgical resection is required to make the diagnosis (19).

Statistical analyses were performed using R statistical software version 3.2.3 (https://www.r-project.org). Continuous variables were compared using T test, categorical variables were compared using Fisher exact test. All confidence intervals are 2-sided 95% CIs and were computed using the exact binomial test. Multiple hypotheses were corrected using Benjamini-Hochberg Procedure.

## Results

### TSHR prevalence

**Figure 1:**
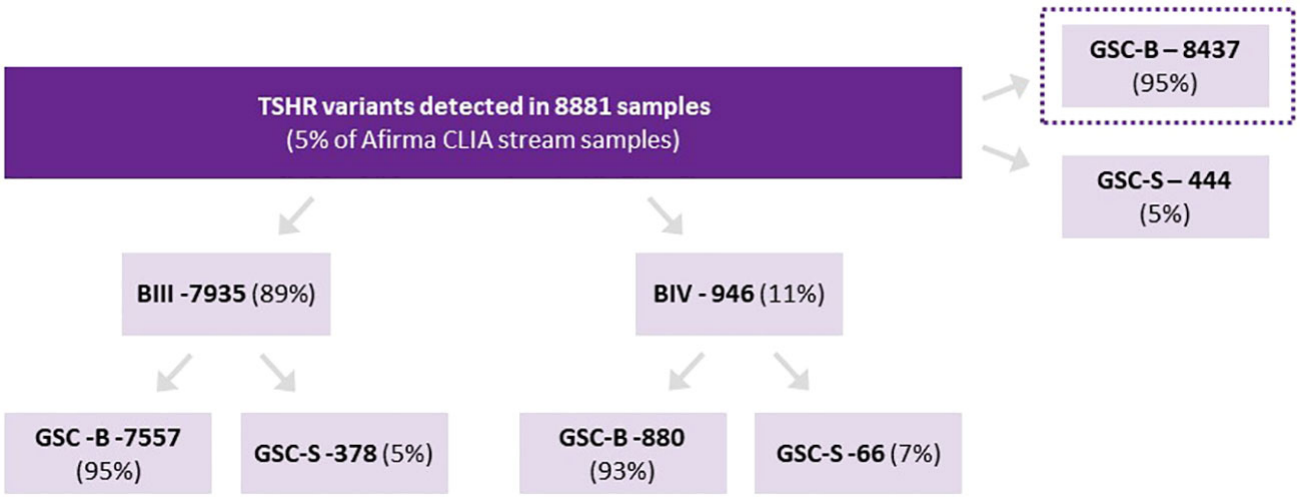
Proportion of TSHR variants assigned as Afirma GSC-B or GSC-S as well as the breakdown of ITN Bethesda categories and Afirma result for each cytology type.

There were 8881 Afirma GSC samples of ITN (Bethesda III/IV only) with isolated TSHR variants. This represents 5.4% of all samples collected over the study period. Of these nodules, 8,437 were categorized as GSC-B and 444 as GSC-S (GSC-S = 5.0% [95% CI 4.6%-5.5%]) (**Figure 1**). Among TSHR mutated nodules that were GSC-S, Bethesda III cytology represented 378/7935 (4.8%) and Bethesda IV 66/946 (7.0%) (p<0.01). There were no specific TSHR codon changes with statistically higher GSC-B vs GSC-S results nor were any subdomains (transmembrane vs intracellular vs extracellular) of the TSHR overrepresented as GSC-B or GSC-S.

In females, 370/7807 (4.7%) ITNs with TSHR variants were GSC-S and in males, 74/1072 (6.9%) were GSC-S, which was statistically higher (p<0.01) (2 samples did not have gender stated on the test request form and were excluded). Age younger than 55 years also had a statistically higher rate of GSC-S TSH variants than age > 55 years 215/3695 (5.8%) and <55 years 229/5186 (4.4%) respectively (p<0.01) (**Table 1**).

**Table 1:** Proportion of TSHR variant distribution by gender and age amongst Afirma GSC-B or GSC-S results.

	GSC				
Excluded 2 without gender	Benign	Suspicious	Total	%GSC-S	P = 0.003
Female	7437	370	7807	4.7%	
Male	998	74	1072	6.9%	
Total	3435	444	3379	5.0%	
	GSC				
Age group	Benign	Suspicious	Total	%GSC-S	P = 0.003
< 55	3480	215	3695	5.8%	
≥55	4957	229	5186	4.4%	
Total	3437	444	8331	5.0%	

### TSHR I568T prevalence and ROM

**Figure 2:**
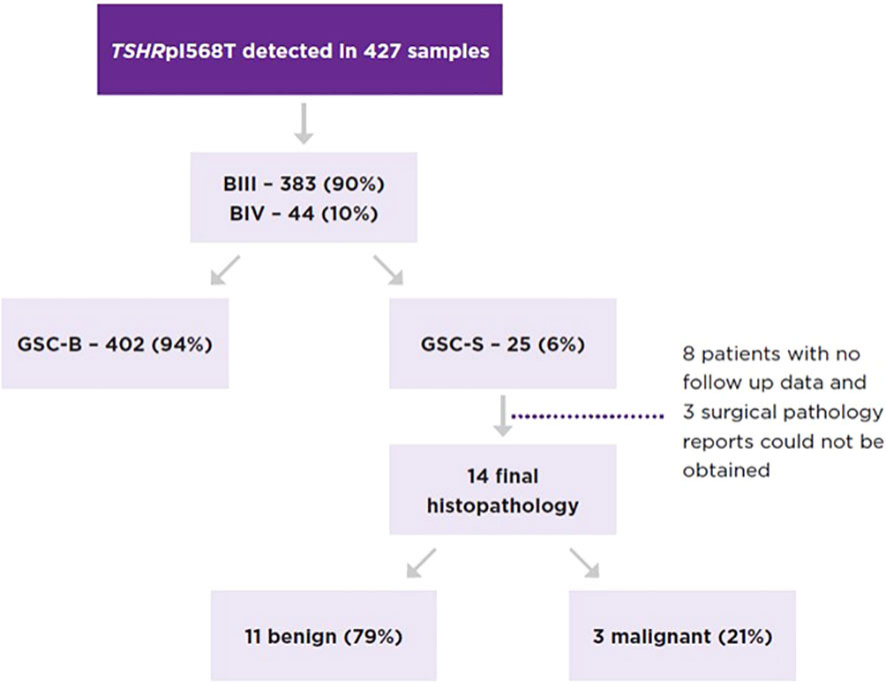
Proportion of TSHRpI568T mutated nodules by Bethesda category and Afirma GSC category as well as the malignancy rate of GSC-S nodules.

A total of 427 ITN samples harbored the TSH I568T variant, 383 from BIII (89.7%) and 44 from BIV (10.3%) cytology. GSC-S was resulted in 17/383 BIII (4.4%) and 8/44 BIV nodules (18.2%) (p<0.01) (**Figure 2**). Therefore, 402 samples (94%) were categorized as GSC-B. Of the 25 GSC-S samples, 14 final histopathology reports were obtained (8 patients had no follow up data or record of surgery and 3 pathology reports could not be obtained). Final pathology showed 11 benign results which were (when described) comprised of follicular adenomas, one Hurthle cell adenoma and one infarcted nodule; and 3 malignancies indicating a 21.4% risk of malignancy [95% CI 4.7-50.8%]. The tumors included a 1.2 cm NIFTP, a 2.0 cm primary infiltrative follicular variant of papillary thyroid carcinoma (FV-PTC) that was multifocal, and a 0.8 cm PTC (**Table 2**). If we make the extreme assumption that the 11 GSC-S samples without histopathology are all benign or malignant, the malignancy risk ranges from 3/25 (12%) to 14/25 (56%). When assuming all GSC-B nodules are true negatives lesions and excluding the 11 GSC-S nodules for which histopathology reports were not obtained, the risk of malignancy in the overall TSHR I568T mutated ITN population is 3/416 (0.72% [95% CI 0.15-2.1%]).

**Table 2:** Patient and tumor characteristics of malignancies discovered within Afirma GSC-S TSHR variants.

Mutation	Age	Gender	Cytology	Nodule Size (cm)	Tumor
I568T	54	F	III	1.1	1.2 cm NIFTP
I568T	72	F	III	3.5	2.0 cm FV-PTC, infiltrative and multifocal
I568T	56	M	III	2.8	0.8 cm PTC
F631V	37	F	III	1.4	1.1 cm PTC
F631L	34	F	III	1.1	1.1 cm NIFTP
T632I	64	F	III	3.6	3.6 cm FTC
M453T	25	F	III	2	1.4 cm minimally invasive FTC
M453T	66	M	IV	2.7	1.0 cm PTC
T632I	29	F	IV	2.7	2.4 cm FTC
TM453T	87	F	III	4.4	1.5 cm FTC, minimally invasive
D633H	35	M	IV	1.6	1.2 cm PTC
M453T	76	F	III	1.4	0.6 cm FV-PTC
I486F	52	F	III	1.5	2.4 cm HCC, minimally invasive

### TSHR variant ROM in GSC-S ITN

**Figure 3:**
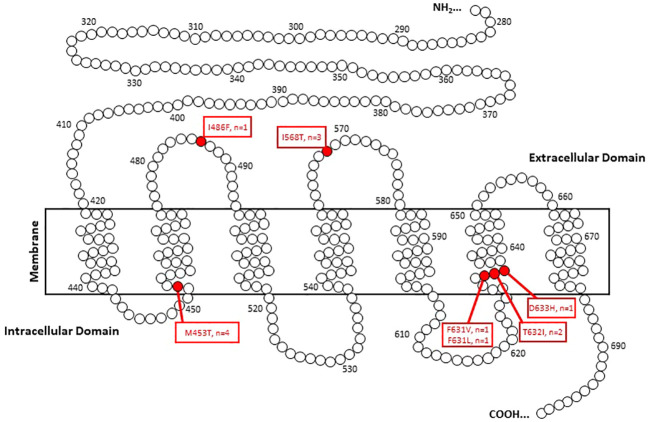
TSHR codon variants associated with malignancies in Afirma GSC-S thyroid nodules.

To assess the ROM of TSHR variants that were not mutations at the 568 codon, 43 pathology reports were obtained where 7 malignancies were discovered (7/43 = 16.3% [95% CI 6.8%-30.7%]). These malignancies included a 1.1cm classical PTC, a 1.1 cm NIFTP, a 3.6 cm widely invasive Follicular Thyroid Carcinoma (FTC), a 1.4 cm minimally invasive FTC, a 1.0 cm PTC with multifocality, a 2.4 cm FTC and a 2.4 cm minimally invasive Hurthle cell cancer with an associated 0.1 cm PTC (**Table 2**). All but one malignancy in this cohort were associated with transmembrane subdomain codon variants at codons 631, 632, and 453 (**Figure 3**). Therefore, 28 more pathology reports from GSC-S nodules with TSHR variants in transmembrane domain codons were obtained from the primary study cohort to see if there was enrichment of malignancy at these receptor locations. This analysis revealed 3/28 (10.7% [95% CI 2.3-28.2%]) malignancies and included a 1.5 cm minimally invasive FTC, a 1.2 cm PTC with oncocytic cell changes, and a 0.6 cm FV-PTC (**Table 2** and **Figure 3**). Benign histology (when reported) included follicular adenomas, Hurthle cell adenomas, multinodular hyperplasia, hyperplastic nodules with hashimoto’s thyroiditis, and one colloid nodule.

When assessing the overall cohort for which pathology reports were available, there were 13/85 malignancies within the GSC-S result of ITNs (15.3% [95% CI 8.4-24.7%]). There was no significant difference in malignant vs benign samples with respect to nodule size, age, or gender. The median nodule size was 2.3 cm [0.9, 6.6], 2.0 cm for malignant and 2.3 cm for benign. The median age was 58 years [20, 91], 47 years for malignant and 59 years from benign. If we assume all GSC-B samples are true negatives, the ROM by detection of a TSHR variant alone is <1.5%.

## Discussion

In the present study, we have investigated the epidemiology of TSHR variants in ITN submitted for molecular analysis by the Afirma GSC. Additionally, the malignancy rate in 85 GSC-S ITN with TSHR variants was assessed which represents the largest cohort published to date. We have demonstrated that the prevalence of isolated TSHR variants in ITN is 5%, similar to other published rates (4, 11). A very small subset of nodules was co-mutated with better characterized thyroid cancer driver mutations from the RAS family and BRAFV600E as previously described, and they were excluded from this analysis in favor of analyzing the risk of malignancy in ITN with isolated TSHR variants as detected by the Afirma XA (11, 20). We also show that Afirma only categorizes 5% of TSHR mutated ITNs as GSC-S. Though there are statistically significant differences shown for GSC-S rates of TSHR mutated ITNs by Bethesda cytology category (IV>III), gender (M>F), and age (more frequent in those under 55 years), they are likely not clinically significant differences to confidently risk stratify patients. Additionally, there does not seem to be a specific codon or subdomain region of the TSHR mutation that consistently predicts malignancy.

This study was inspired by a query after the management of an aggressive case of PTC where the index lesion was positive for a TSHRpI568T mutation (12). We showed that this variant is extremely rare, representing only ~0.7% of all molecular findings in the Afirma database. Additionally, the overall risk of malignancy in nodules harboring TSHRpI568T mutations is very low (< 2%) when analyzed by its presence alone. However, when categorized as GSC-S, the risk of malignancy has a point estimate of 21% based on a limited number of pathology reports. This observation highlights the value of the Afirma GSC to significantly alter the risk of malignancy in ITNs with its benign vs suspicious categorization, even in the presence of low-risk mutations.

As shown in **Table 2**, not all malignancies associated with TSHR mutated ITN are low risk. Cancers reported include widely invasive FTC and a 2 cm infiltrative FV-PTC with multifocality. It is unclear if there were clinical indicators of these more aggressive tumors pre-operatively. One of the limitations of this study is not having the clinical and ultrasound findings available to know how TSH level, ATA or TIRADs ultrasound risk stratification or how individual patient risk factors may have influenced the decision to monitor or refer patients for surgery (21–23). Another limitation of this study is the assumption that all GSC-B TSHR mutated ITN are true negatives. Given the 96% negative predictive value of an Afirma GSC-B result and the known overall low risk TSHR mutated ITN, it is very unlikely there are many false negative TSHR mutated thyroid nodules (15).

The only other commercially available thyroid nodule molecular diagnostic test in the United States that reports TSHR variants is the Thyroseq Thyroid Genomic Classifier (Thyroseq) (24). Thyroseq is a targeted next generation sequencing test whose current iteration (v3) evaluates point mutations, gene fusions, copy number alterations and abnormal gene expression in 112 thyroid cancer related genes (24). Thyroseq reports TSHR mutations as “currently negative” with a <10% probability of a low-risk cancer or NIFTP and a recommendation of active surveillance (25). An exhaustive literature search to ascertain the ROM in operated TSHR mutated ITNs reveals 3/29 malignancies reported for a point estimate of 10% cancer rate (4, 11, 26–35). Almost all these studies utilized the Thyroseq platform, thus it is reasonable to assume there were other concerning clinical or ultrasound features prompting a recommendation for surgery given the low risk ascribed to TSHR mutations. The allelic frequency of the TSHR variant may influence malignancy risk. Mon et al. describe nodules with isolated TSHR variants and < 36% allelic frequency as all being histologically benign. However, among 5 ITN with allelic frequency between 36-45%, 3 were FTC and 2 were benign, indicating a poor discrimination of malignancy risk at this allelic frequency range (11).

Based on this largest survey of TSHR mutated ITNs, it is true that the overall malignancy risk is low. Only 5% of ITN undergoing Afirma testing harbor a TSHR variant and only 5% of that subset are categorized as GSC-S. The Afirma GSC’s ability to assign a benign or suspicious call provides value for risk stratifying patients with this low-risk mutation (15). As opposed to a reported risk of < 10% with a recommendation for active surveillance, Afirma will call 95% of TSHR mutated ITNs as GSC-B, with an extrapolated ≤= 4% risk of malignancy, and likely lower based on the known overall low risk of TSHR mutated nodules. Therefore, these patients can be monitored conservatively, like those with a Bethesda II cytology result (18). Alternatively, when assigned a GSC-S result, the risk of malignancy is >= 15%, a clinically significant difference that likely leads to a very different shared decision-making conversation with patients. At this relatively low risk of malignancy, dependent on clinical and ultrasound features, some may be comfortable with an active surveillance approach. Given this risk is similar to the baseline risk of an ITN, many may feel a diagnostic lobectomy is more appropriate, which may also be adequately therapeutic. If a TSHR variant is detected by molecular testing, the above clinical decisions should be made in the context of thyroid stimulating hormone (TSH) testing (if not done prior to nodule FNA) and assessment of thyroid autonomy when the TSH is suppressed.

Population based risk assessments of thyroid nodule mutations provide beneficial guidance for a general approach to patients with ITN and thyroid cancer. However, physicians treat individuals, and this study demonstrates the utility of the Afirma GSC to risk stratify patients with TSHR mutated ITN in a clinically meaningful way. An Afirma GSC-B was reported for nearly all nodules with an isolated TSHR variant, and they likely avoided diagnostic surgery. Conversely, a minority of patients with an isolated TSHR variant received an Afirma GSC-S result. An Afirma GSC-S result is the foundation that informs further risk assessment based on XA results and may optimize the approach to initial therapy in patients with ITN (13).

## Data availability statement

The datasets presented in this article are not readily available because TSHR variant sequence information is proprietary information of Veracyte, Inc. Requests to access the datasets should be directed to joshua.klopper@veracyte.com.

## Ethics statement

Ethical review and approval was not required for the study on human participants in accordance with the local legislation and institutional requirements. Written informed consent for participation was not required for this study in accordance with the national legislation and the institutional requirements.

## Author contributions

JPK, RTK, SW, YH, JH and GCK designed the study. DW, JEP, SH, BO’D, JN, DJ, AC and JS review clinical records and collected data. JPK, SW, and YH prepared the manuscript. All authors reviewed the manuscript. JPK, DW, RTK, JS and YH revised the manuscript. All authors contributed to the article and approved the submitted version.
